# Validity of the Inventory of Depressive Symptomatology Self-Report (IDS-SR) in Older Adults With Subjective Cognitive Decline (SCD) and Mild Cognitive Impairment (MCI)

**DOI:** 10.1186/s40359-026-04514-5

**Published:** 2026-05-11

**Authors:** Jingru Wang, Meirong Chen, Yanping Jin, Xiangqing Xie, Yun Yang, Lin Huang, Liang Cui, Huizhi Ma, Jun Ma, Qihao Guo, Yingzi He

**Affiliations:** 1 Department of Gerontology, Shanghai Sixth People’s Hospital Affiliated to Shanghai Jiao Tong University School of Medicine, Shanghai, China; 2 Department of Nursing, Shanghai Sixth People’s Hospital Affiliated to Shanghai Jiao Tong University School of Medicine, Shanghai, China; 3 Jiangwan Hospital of Hongkou District, Shanghai, China

**Keywords:** IDS-SR, Subjective cognitive decline, Mild cognitive impairment, Validity, Reliability

## Abstract

**Background:**

This study aims to validate the suitability of the Inventory of Depressive Symptomatology Self-Report (IDS-SR) for evaluating mood disorders in older adults with subjective cognitive decline (SCD) and mild cognitive impairment (MCI).

**Methods:**

A total of 1670 participants aged ≥ 50 years were included (531 males; mean age 65.79 ± 7.39 years). Construct validity of the IDS-SR was evaluated using exploratory factor analysis (EFA) and confirmatory factor analysis (CFA). Criterion validity was examined using Pearson correlation analyses between IDS-SR scores and scores on the Hamilton Depression Scale (HAMD) and the Hamilton Anxiety Scale (HAMA). Receiver operating characteristic (ROC) curve analysis was conducted to determine the ability of the IDS-SR and its subscales to distinguish mood disorders in SCD and MCI. Cronbach’s α and the intraclass correlation coefficient (ICC) were employed to assess the reliability of the scale.

**Results:**

The EFA demonstrated that the major factors explained 47.722% of the total variance. The CFA confirmed a three-factor model of the IDS-SR and indicated good construct validity. The fit indices were as follows: chi-square/degree of freedom (*χ*^2^/*df*) = 3.316, root mean square error of approximation (RMSEA) = 0.054, goodness of fit index (GFI) = 0.945, adjusted goodness-of-fit index (AGFI) = 0.928, comparative fit index (CFI) = 0.921, incremental fit index (IFI) = 0.922, and Tucker-Lewis index (TLI) = 0.908. The total Cronbach’s α was 0.839. The ICC for the total scale was 0.952.

**Conclusions:**

The IDS-SR scale is a reliable and valid screening tool for detecting mood disorders in older adults with SCD and MCI. This study provides the first validation of the scale in this population.

**Supplementary Information:**

The online version contains supplementary material available at https://doi.org/10.1186/s40359-026-04514-5.

## Introduction

 Subjective cognitive decline (SCD) and mild cognitive impairment (MCI) are two key stages that precede the onset of dementia [1]. SCD refers to a self-perceived decline in cognitive function despite normal performance on objective tests [2]. In contrast, MCI is defined by measurable cognitive deficits that do not meet the criteria for dementia [3]. More evidence shows that individuals with SCD or MCI exhibit significantly higher levels of depression and anxiety compared to healthy controls [4, 5]. Among those with MCI, comorbid depression and/or anxiety are associated with a higher risk of progression to dementia and with faster cognitive decline [6]. However, findings on how mood disorders affect the progression from SCD to objective cognitive impairment remain inconsistent. Some studies suggest that SCD and anxiety independently increase the risk of MCI and dementia. Their co-occurrence further elevates this risk and accelerates disease progression [7]. In memory clinic samples of SCD, high levels of depressive symptoms are also linked to faster cognitive decline during follow-up. These findings suggest that depression may independently accelerate the transition to objective cognitive impairment [8]. In contrast, other studies indicate that anxiety, rather than depression, may play a more prominent role in this process [9]. Overall, these findings suggest that depression and anxiety are not merely comorbid conditions but may actively contribute to cognitive deterioration [10]. This topic has received increasing attention in recent years. The number of publications on mood symptoms in cognitive aging has more than doubled over the past ten years [11]. Therefore, because SCD and MCI represent early stages of dementia, it is essential to assess mood symptoms in these populations for both clinical practice and research.

Several instruments are commonly used to assess mood symptoms, including the Hamilton Rating Scale for Depression (HRSD), the Hamilton Depression Rating Scale (HAMD), the Hamilton Anxiety Scale (HAMA), and the Geriatric Depression Scale (GDS). Among them, the HRSD is widely used to evaluate the severity of depression [12]. However, its core versions fail to fully cover the diagnostic criteria for major depressive disorder defined in the Diagnostic and Statistical Manual of Mental Disorders, Fourth Edition (DSM IV) [13]. The HAMD and HAMA are also widely used in clinical practice. However, both require trained assessors to conduct structured interviews and clinical observations [14, 15]. This process is time-consuming. Similarly, the Cornell Scale for Depression in Dementia (CSDD) is an interviewer-administered instrument. It relies on caregivers reports and a clinical judgment [16]. It is particularly useful for assessing depression in older adults with dementia [17]. In contrast, self-report scales are more time-efficient and easier to administer. Nevertheless, some self-report tools, such as the GDS, mainly assess affective and cognitive symptoms. They provide limited coverage of somatic symptoms (e.g., energy, sleep, and appetite) [18].

The Inventory of Depressive Symptomatology Self-Report (IDS-SR) is a widely used self-report instrument that aligns with DSM criteria. It is designed for screening and assessing depression severity [19, 20]. The scale includes 30 items and yields a total score from 0 to 84. A cutoff score of ≥ 18 on the IDS-SR indicates clinically relevant depressive symptoms [20]. The IDS-SR assesses not only typical depressive and anxiety symptoms but also atypical manifestations. Its factor structure includes three dimensions: “mood/cognition”, “anxiety/somatic”, and “sleep” [20, 21]. Each dimension can function as a separate subscale to assess the severity of specific symptom clusters. This multidimensional structure distinguishes the IDS-SR from other tools, such as the Centre for Epidemiologic Studies Depression Scale (CES-D), GDS, and HRSD. Previous studies have shown that the IDS-SR has good reliability and validity in both clinical and community samples. It has also been validated for use in older adults [20, 22].

The Quick Inventory of Depressive Symptomatology Self-Report (QIDS-SR), a 16-item short form of the IDS-SR, has gained increasing use due to its focus on the nine core symptom domains of major depressive disorder [23]. However, according to DSM criteria, while core symptoms are necessary for diagnosis, non-core symptoms such as somatic and prodromal features may occur independently and carry clinical significance. Thus, a comprehensive measure like the IDS-SR, which captures both core and non-core dimensions, is particularly important for a full assessment, especially in older adults.

Based on this evidence, we introduce the IDS-SR in individuals with SCD and MCI to improve the assessment of mood symptoms during the early stages of dementia.

## Materials and methods

### Participants

This study was cross-sectional and participants were recruited from the cohort of Chinese Preclinical Alzheimer’s Disease Study (C-PAS). C-PAS is an observational longitudinal study conducted in Shanghai, China, starting in April 2019. The detailed recruitment methods, inclusion and exclusion criteria, and ethical review of the participants can be found in our previous article [24].

### Data collection

In C-PAS, all participants underwent a thorough history-taking, neurological examination, neuropsychological assessment, neuroimaging and laboratory testing. The detailed data collection information can be found in our previous article [24]. In this study, we collected information including demographic information about age, gender, marriage, education, source, cognitive diagnosis, and history of disease. And then there were neuropsychological tests containing IDS-SR, HAHA, HAMD, Addenbrooke’s Cognitive Examination III (ACE-III), and Montreal Cognitive Assessment-Basic (MoCA-B).

### Assessment of depression and anxiety

Mood disorders were assessed using the 30-item IDS-SR (Table S1) [19, 20]. Each item is rated on a 0–3 scale, reflecting increasing symptom severity. The total score (range: 0–84) is derived by summing 28 of 30 items: for the paired items on appetite change and weight change, only the higher-scoring item from each pair is included [20]. The inventory is designed to be completed by the patient in one sitting. For those with reading difficulties, the questionnaire can be verbally administered by a staff member to assist them. Furthermore, the IDS-SR established a three-factor structure comprising “mood/cognition” (items 5, 8, 10–14, 16–22), “anxiety/somatic” (items 6, 7, 9, 15, 23–30), and “sleep” (items 1–4) [20]. We aim to examine the validity, and reliability of the IDS-SR. It has been translated into over 30 dialects including Chinese versions. See www.ids-qids. org for all available translated instruments.

The diagnosis of clinically significant depression and anxiety was established by a psychiatrist. That were based on the HAMD (17 items) and the HAMA (14 items), in accordance with the DSM IV criteria. Notably, previous studies have found that HAMD and HAMA have good valid and reliability [25, 26], supporting their application in the present study. For our analysis, participants were divided into four groups by mood symptoms based on HAMD (17 items) and HAMA (14 items) scores: without depression or anxiety, only depression, only anxiety, and depression with anxiety.

### Assessment of cognitive function

In this study, we focused on individuals with SCD and MCI. MCI was defined using an actuarial neuropsychological method proposed by Edmonds et al. [27]. SCD was defined based on the SCD-plus guidelines of Jessen et al. [28], requiring the presence of self-perceived cognitive decline and related concerns.

### Statistical analysis

All statistical analyses were performed using the SPSS 25.0 edition and Amos 23.0 edition. The descriptive characteristics for categorical variables were summarized as percentages, and significant differences were evaluated using a *χ*^2^ test. Continuous variables were summarized as mean ± SD, and comparisons were performed using the one-way analysis of variance (ANOVA).

#### Validity

The exploratory factor analysis (EFA) and confirmatory factor analysis (CFA) were applied to assess the construct validity of the IDS-SR. EFA was conducted on the full sample (*N* = 1670), while CFA was performed specifically in the MCI group (*N* = 786). In EFA, we calculated Bartlett’s spherical test value and Kaiser-Meyer-Olkin (KMO) test value. When KMO value is higher than 0.6, it is suitable for EFA [29]; however, > 0.8 is more typical. The Bartlett’s spherical test value should be significant (*P* < 0.001). Then, the principal component analysis (PCA) with varimax rotation was performed to extract the factors and obtain the most meaningful original factor structure of the IDS-SR. If the factor loadings of the items are lower than 0.5, it shows that they need to be deleted, but items with dual factor loadings > 0.4 were eliminated from the factor analysis [30]. In CFA, the maximum likelihood confirmatory factor analysis was used to examine the underlying latent variable structure of the IDS-SR. The chi-square/degree of freedom (*χ*^2^/*df*) < 5, root mean square error of approximation (RMSEA) from 0.08 to 0.1, comparative fit index (CFI), Tucker-Lewis index (TLI), and incremental fit index (IFI) > 0.9, goodness of fit index (GFI), and adjusted goodness-of-fit index (AGFI) from 0.8 to 0.9, the that the fit of the model is within an acceptable range [31–34].

Correlation with HAMD and HAMA was used to calculate the criterion validity. To evaluate the discriminative ability of the IDS-SR among SCD and MCI, receiver operating characteristic (ROC) curves were plotted and the area under the curve (AUC) was calculated. The optimal cut-off score for each comparison was determined by maximizing Youden’s index (sensitivity + specificity − 1).

#### Reliability

The internal consistency reliability of the IDS-SR was assessed using Cronbach’s α, with values above 0.6 indicating acceptable reliability [35, 36]. Test-retest reliability over a three-month interval was evaluated using the intraclass correlation coefficient (ICC).

## Results

### Characteristics of the study population

**Fig. 1:**
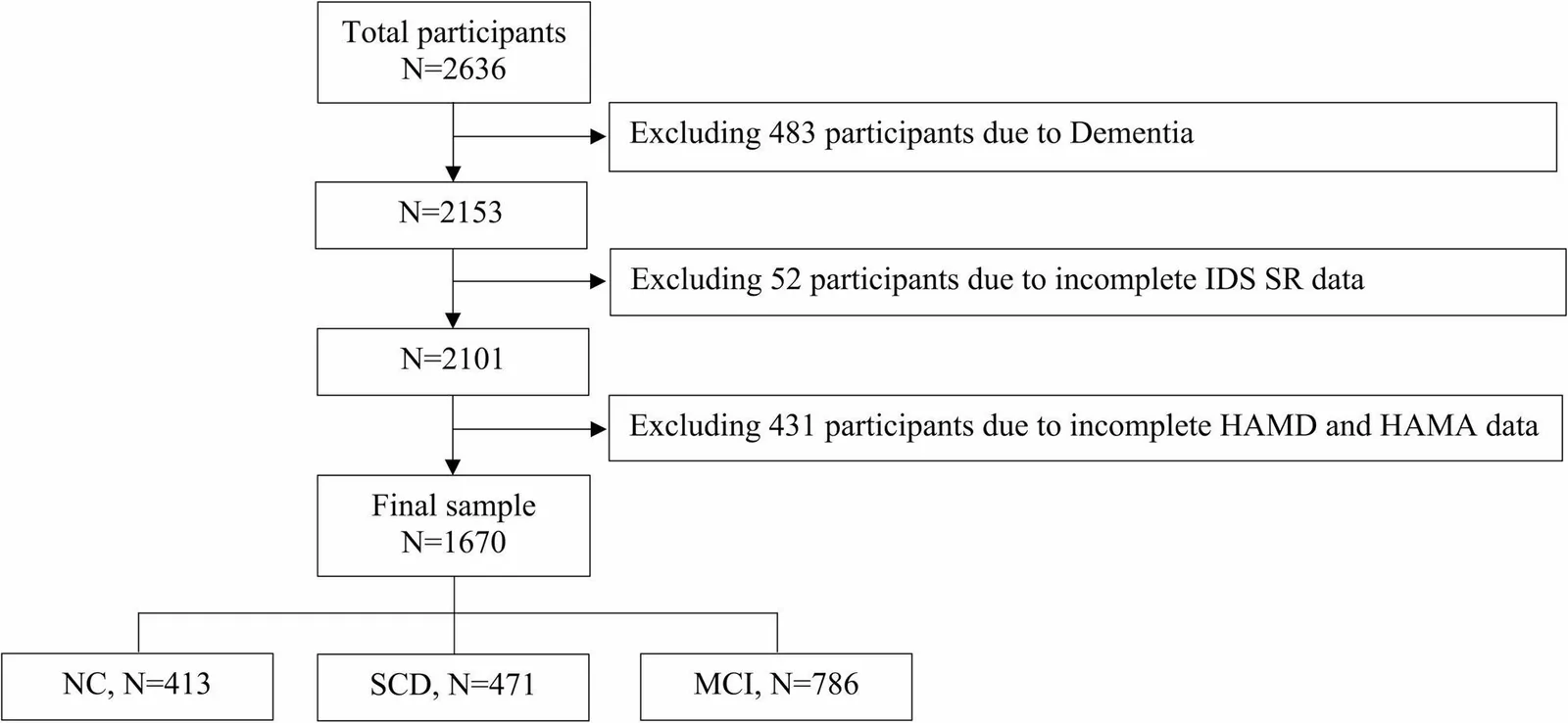
Flow chart of participants enrolled

A total of 2636 participants were enrolled between April 2019 and May 2023. The records were used as baseline data. After excluding cases with missing data on the IDS-SR or cognitive diagnosis, 1670 participants were included in the final analysis (Fig. 1). Among these participants, 31.8% were male and 68.2% were female. The mean age was 65.79 ± 7.39 years. In terms of cognitive status, 413 participants (24.7%) had normal cognition (NC), 471 (28.2%) had SCD, and 786 (47.1%) had MCI.

We used both the HAMD and the IDS-SR to assess depression. The prevalence of depression was 20.6% based on the HAMD and 19.5% based on the IDS-SR (data not shown). Table 1 shows the detailed demographic characteristics and neuropsychological measures of the study population. For demographic variables, we found significant differences among the four groups in gender, marriage status, recruitment source, and hyperlipidemia (all *P* < 0.05). Similarly, we also observed significant differences among the four groups in neuropsychological measures (all *P* < 0.01). Notably, IDS-SR scores were significantly higher in participants with anxiety or depression compared to those without either condition. The highest scores were observed in participants with both depression and anxiety (both *P* < 0.01).

**Table 1:** The Characteristics of participants according to Depression/Anxiety status among all participants (*N*=1670)

Variables	No Depression No Anxiety	Depression	Anxiety	Depression with Anxiety	Test statistics	P
N (%)	1076(64.40)	57(3.40)	250(15.00)	287(17.20)		
Gender (%)					41.445	<0.001
Male	395(36.7)	15(26.3)	71(28.4)	50(17.4)		
Female	681(63.3)	42(73.7)	179(71.6)	237(82.6)		
BMI (kg/m^2^)	23.80±5.90	23.05±3.82	24.15±3.41	24.13±11.34	0.540	0.655
Age (years)	65.62±7.55	65.32±6.59	65.94±6.82	66.36±7.44	0.863	0.460
Marriage (%)					19.269	0.023
Single	14(1.4)	1(1.9)	4(1.7)	4(1.4)		
Married	915(90.1)	44(83.0)	204(87.6)	227(82.2)		
Divorced	29(2.9)	5(9.4)	9(3.9)	17(6.2)		
Widowed	58(5.7)	3(5.7)	16(6.9)	28(10.1)		
Education (%)	12.05±3.35	11.57±3.35	11.55±3.24	11.45±3.11	3.530	0.014
APOE carrier (%)	91(23.3)	6(26.1)	18(19.8)	23(20.4)	0.983	0.805
Source (%)					31.856	<0.001
Outpatient department	269(26.6)	26(49.1)	73(30.7)	113(40.9)		
Be referred	388(38.4)	18(34.0)	91(38.2)	86(31.2)		
Voluntary counseling	353(35.0)	9(17.0)	74(31.1)	77(27.9)		
Hypertension (%)	382(38.4)	18(34.0)	93(40.3)	105(38.0)	0.789	0.852
Hyperlipidemia (%)	189(19.0)	14(26.4)	67(29.0)	72(26.1)	15.054	0.002
Diabetes (%)	150(15.1)	12(22.6)	37(16.0)	47(17.0)	2.557	0.465
ACE-III score	75.96±9.73	72.41±13.68	74.21±9.17	73.67±9.67	6.797	<0.001
MoCA-B score	23.89±3.79	22.84±5.35	23.57±3.70	23.37±3.80	2.644	0.048
IDS-SR score	7.84±6.53	14.53±9.75	12.77±7.46	21.11±9.66	251.841	<0.001
Mood/cognition score	2.37±2.80	4.63±4.13	3.62±3.42	6.87±5.02	133.695	<0.001
Anxiety/somatic score	3.12±3.15	5.42±4.61	5.50±3.42	8.60±4.17	201.480	<0.001
Sleep score	2.23±2.16	4.30±2.50	3.46±2.26	5.25±2.65	143.760	<0.001

Continuous variables are presented as mean ± SD and categorical variables as number (percentage). Differences between two groups were analysed by Chi-square tests for categorical variables and one-way analysis of variance for continuous variables

*Abbreviations NC* normal cognition, *BMI* body mass index, *ACE-III * Addenbrooke’s Cognitive Examination-III, *MoCA-B* Montreal Cognitive Assessment-Basic, *IDS-SR* Inventory of Depressive Symptomatology Self-Report, *SCD* subjective cognitive decline, *MCI* mild cognitive impairment

### Validity

#### Construct validity

In the EFA, the KMO value was 0.91, and Bartlett’s test of sphericity was significant (*P* < 0.001). These results indicate that the data were suitable for factor analysis. We used PCA for factor extraction and varimax rotation. We retained factors with eigenvalues greater than 1. Three factors were extracted, with a cumulative variance of 47.722%. The factors were defined as follows: mood/cognition (items 5, 8, 10, 16, 17, 19, 21, 24), anxiety/somatic (items 20, 23, 25, 26, 27, 30), and sleep (items 1, 2, 3).

**Fig. 2:**
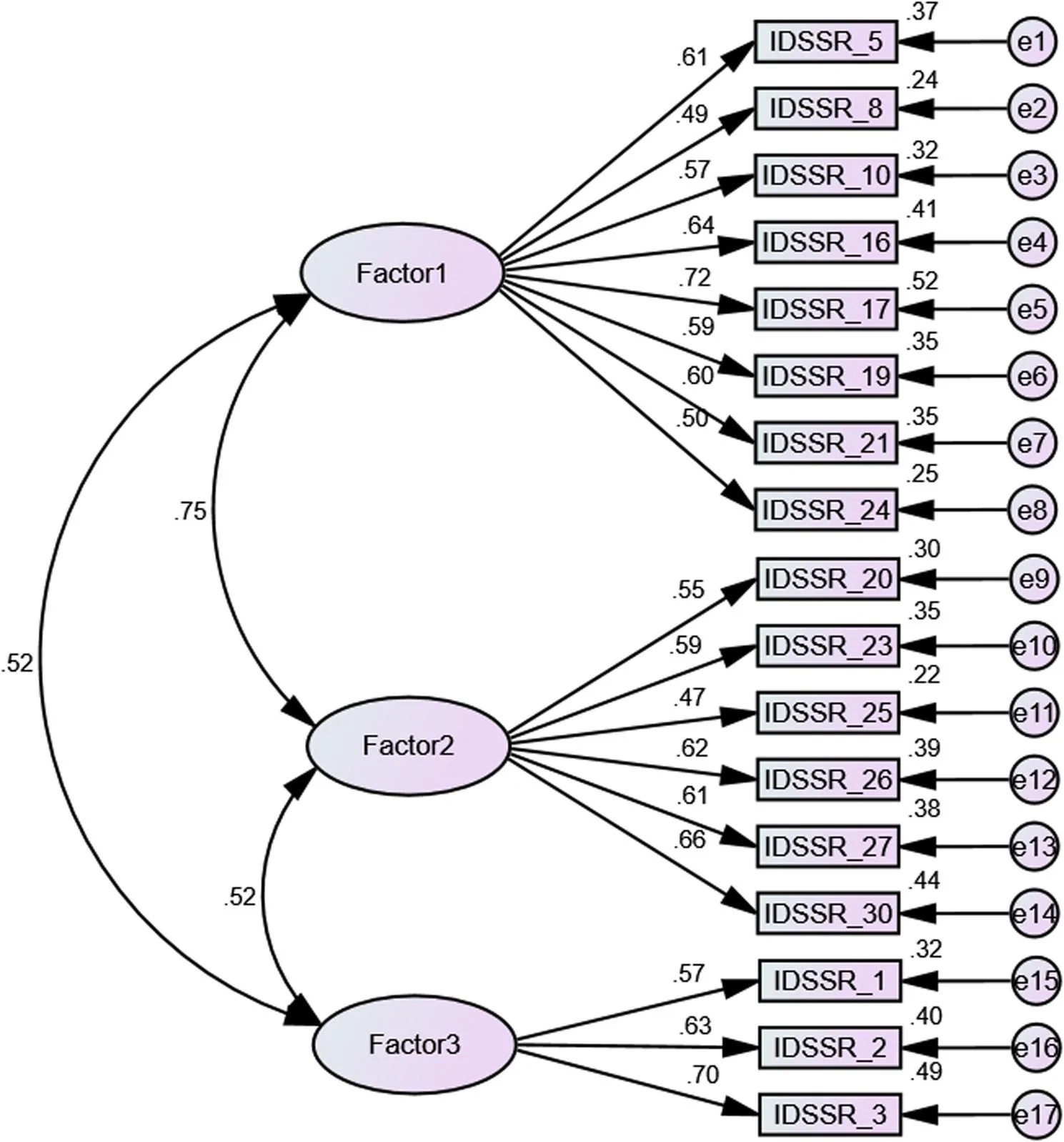
Three factor model of the IDS-SR (N = 786). Note: Factor1: Mood/cognition; Factor2: Anxiety/somatic; Factor3: Sleep, IDS-SR: Inventory of Depressive Symptomatology Self-Report

The CFA showed the three-factor model fit the data well. The fit indices were as follows: *χ*^2^/*df* = 3.316, RMSEA = 0.054, GFI = 0.945, AGFI = 0.928, CFI = 0.921, IFI = 0.922, TLI = 0.908. All indices met the recommended thresholds. The factor loadings of the variables are shown in Fig. 2.

#### Criterion validity

To examine the criterion validity of the IDS-SR, we analyzed its correlations with the HAMD and HAMA (Table 2). Both the total score and its factor scores were significantly and positively correlated with HAMD and HAMA scores. These results indicate that the IDS-SR has good criterion validity.

**Table 2:** The correlation among the IDS-SR scores and cognitive function, HAMD, and HAMA scores (*N* = 1670)

Clinical diagnosis	IDS SR scores	MoCA-B	ACE-III	HAMD	HAMA
NC	Mood/cognition score	-0.194**	-0.213**	0.528**	0.460**
	Anxiety/somatic score	-0.098*	-0.138**	0.594**	0.589**
	Sleep score	-0.054	-0.117*	0.550**	0.471**
	Total score	-0.153**	-0.196**	0.675**	0.618**
SCD	Mood/cognition score	-0.125**	-0.146**	0.502**	0.495**
	Anxiety/somatic score	-0.011	-0.071	0.523**	0.571**
	Sleep score	-0.079	-0.087	0.532**	0.449**
	Total score	-0.089	-0.124**	0.614**	0.612**
MCI	Mood/cognition score	-0.061	-0.084*	0.487**	0.434**
	Anxiety/somatic score	-0.028	-0.081*	0.532**	0.527**
	Sleep score	-0.024	-0.037	0.462**	0.397**
	Total score	-0.045	-0.083*	0.588**	0.543**

*Abbreviations IDS-SR * Inventory of Depressive Symptomatology Self-Report, *NC* normal cognition, *SCD* subjective cognitive decline, *MCI * mild cognitive impairment, *ACE-III* Addenbrooke’s Cognitive Examination-III, *MOCA-B* Montreal Cognitive Assessment-Basic, *HAMA* Hamilton Anxiety Scale, *HAMD* Hamilton Depression Rating Scale

* *P*< 0.05; ** *P*< 0.01

We also examined the relationships between the IDS-SR and cognitive function measures (ACE-III, MoCA-B) (Table 2). In participants with NC, the total IDS-SR score was negatively correlated with both the MoCA-B (*R* = -0.153, *P* < 0.01) and ACE-III scores (*R* = -0.196, *P* < 0.01). In participants with SCD and MCI, the total IDS-SR score remained negatively correlated with ACE-III scores, but its correlation with MOCA-B scores was no longer significant.

#### ROC analysis

ROC analyses were conducted to assess the ability of IDS-SR to distinguish individuals with depression, anxiety, or both from those without these conditions within each cognitive subgroup (Table 3). In the SCD subgroup, the IDS-SR cutoff scores were 10.5 for depression (sensitivity 61.1%, specificity 72.2%), 8.5 for anxiety (sensitivity 71.4%, specificity 57.9%), and 12.5 for depression with anxiety (sensitivity 83.3%, specificity 80.8%). The corresponding AUC values were 0.669 (95% confidence interval [CI]: 0.539-0.800), 0.698 (95% CI: 0.637–0.759), and 0.894 (95% CI: 0.860–0.929), respectively. In the MCI subgroup, the IDS-SR cutoff scores were 9.5 for depression (sensitivity 71.4%, specificity 68.3%), 7.5 for anxiety (sensitivity 73.9%, specificity 56.2%), and 14.5 for depression with anxiety (sensitivity 74.8%, specificity 86.5%). The corresponding AUC values were 0.763 (95% CI: 0.679–0.848), 0.688 (95% CI: 0.636–0.739), and 0.867 (95% CI: 0.832–0.901), respectively.

**Table 3:** ROC analyses for IDS-SR score to differentiate individuals with Depression, Anxiety, Depression with Anxiety

Group	Variables	AUC	Cut-off	sensitivity	specificity	95% Confidence Interval	*P*
All	Depression	0.739	10.5	0.632	0.742	(0.675, 0.804)	< 0.001
	Anxiety	0.717	7.5	0.760	0.565	(0.683, 0.751)	< 0.001
	Depression with Anxiety	0.887	12.5	0.815	0.819	(0.865, 0.908)	< 0.001
NC	Depression	0.772	12.5	0.636	0.863	(0.635, 0.908)	0.002
	Anxiety	0.799	9.5	0.775	0.719	(0.727, 0.871)	< 0.001
	Depression with Anxiety	0.911	12.5	0.821	0.863	(0.872, 0.950)	< 0.001
SCD	Depression	0.669	10.5	0.611	0.722	(0.539, 0.800)	0.016
	Anxiety	0.698	8.5	0.714	0.579	(0.637, 0.759)	< 0.001
	Depression with Anxiety	0.894	12.5	0.833	0.808	(0.860, 0.929)	< 0.001
MCI	Depression	0.763	9.5	0.714	0.683	(0.679, 0.848)	< 0.001
	Anxiety	0.688	7.5	0.739	0.562	(0.636, 0.739)	< 0.001
	Depression with Anxiety	0.867	14.5	0.748	0.865	(0.832, 0.901)	< 0.001

*Abbreviations IDS-SR* Inventory of Depressive Symptomatology Self-Report, *NC* normal cognition, *SCD * subjective cognitive decline, *MCI* mild cognitive impairment, *AUC* area under the curve

* compared with No Depression No Anxiety group, *P* < 0.05

** compared with No Depression No Anxiety group, *P* < 0.01

**Fig. 3:**
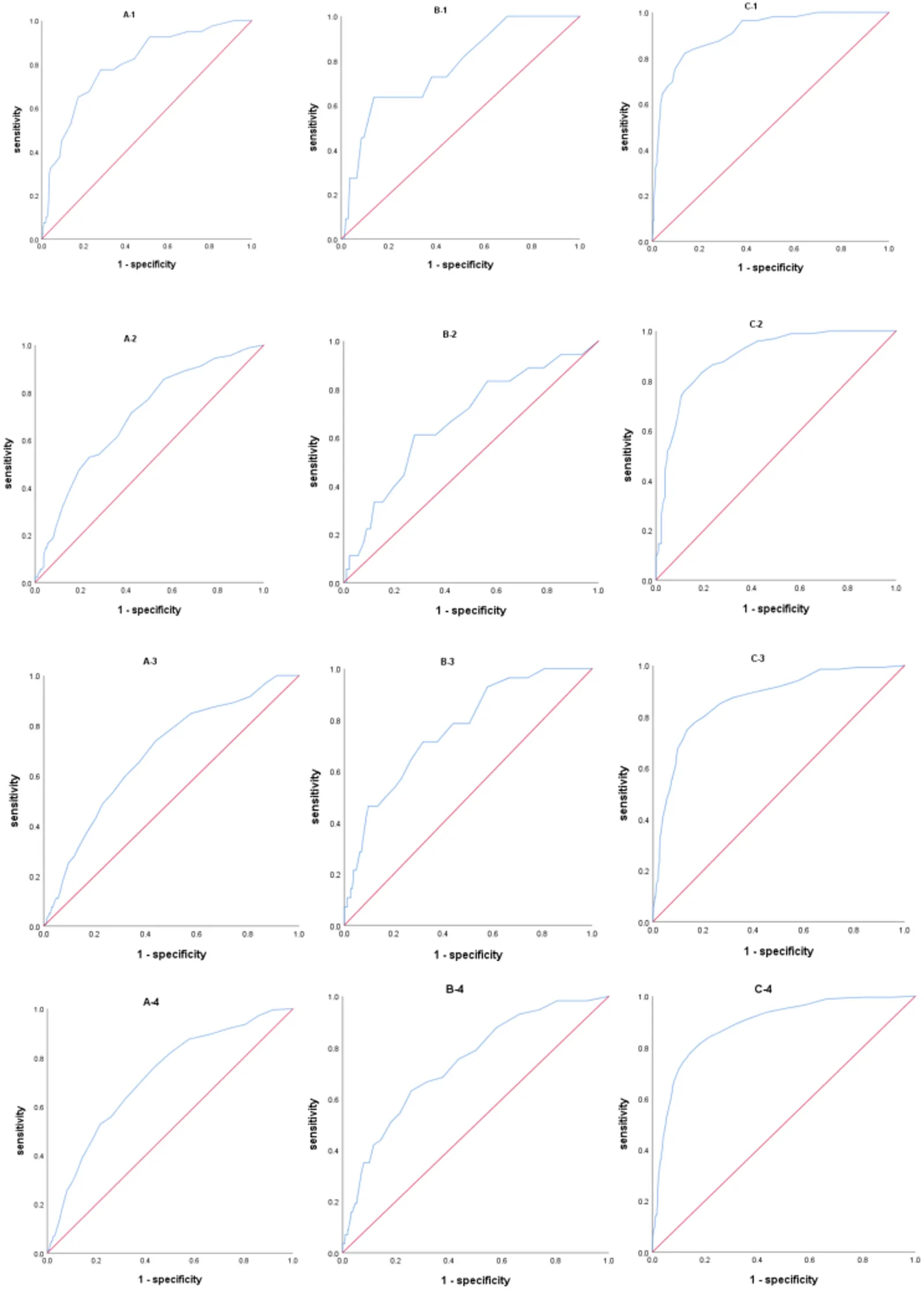
ROC curves for IDS-SR in the identification of anxiety (vs normal) (A-1. in NC participants; A-2. in SCD participants; A-3. in MCI participants; A-4. in all participants), depression (vs normal) (B-1. in NC participants; B-2. in SCD participants; B-3. in MCI participants; B-4. in all participants), depression with anxiety (vs normal) (C-1. in NC participants; C-2. in SCD participants; C-3. in MCI participants; C-4. in all participants)

The comparison of ROC curves was conducted within each cognitive subgroup (Fig. 3). No significant differences were observed between depression and anxiety in any subgroup. However, the AUC values for both depression and anxiety were significantly lower than those for depression with anxiety. Overall, as the number of mood disorders increased, the IDS-SR AUC showed an upward trend. For the identification of anxiety alone and depression with anxiety, greater cognitive impairment was associated with a declining trend in the AUC of IDS-SR. Notably, this pattern was not observed for the depression alone, where the highest AUC occurred in participants with NC.

### Reliability

In Table 4, the IDS-SR demonstrated good internal consistency, with a total Cronbach’s α of 0.839. The subscale alphas were 0.786 for mood/cognition, 0.764 for anxiety/somatic, and 0.670 for sleep. Test-retest reliability was also high, with an ICC of 0.952 for the total scale. The ICCs for the subscales were 0.879 for mood/cognition, 0.881 for anxiety/somatic, and 0.860 for sleep.

**Table 4:** Reliability analysis of IDS-SR

Domains	Cronbach’s α	Intraclass Correlation Coefficient
Factor 1: Mood/cognition	0.786	0.879
Factor 2: Anxiety/Somatic	0.764	0.881
Factor 3: Sleep	0.670	0.860
Total: IDS-SR	0.839	0.952

*Abbreviations IDS-SR* Inventory of Depressive Symptomatology Self-Report

## Discussion

This study aimed to validate the psychometric properties of the IDS-SR for evaluating mood disorders in patients with SCD and MCI. The results confirmed that the IDS-SR is a reliable and valid instrument for this population, with particularly strong performance in identifying depression with anxiety.

In this study, EFA identified a three-factor structure comprising mood/cognition, anxiety/somatic, and sleep among participants with SCD and MCI. The structure was further validated in the MCI group. CFA indicated that the three-factor model achieved a good fit to the data. This structure is highly consistent with the three-factor model of the IDS-SR originally reported by Rush et al. [20]. Subsequent studies have also confirmed its stability across different cultural contexts [21, 22]. Some differences in item composition were observed. Rush et al. [20] included items 1 to 4 (initial insomnia, middle insomnia, early morning awakening, and sleep too much) in in the sleep factor for patients with major depressive disorder, whereas our sleep factor comprised only items 1 to 3 (initial insomnia, middle insomnia, and early morning awakening). Similarly, the Arjadi R et al. [22] classified item 15 (concentration/decision-making) under the anxiety/somatic factor in a community-dwelling population. In our study, this item was excluded due to low factor loading. These discrepancies may be attributable to differences in sample characteristics and variations in the statistical methods.

Notably, Hegeman et al. [37] reported that in older adults with major depressive disorder, sleep items 1 to 3 (initial insomnia, middle insomnia, and early morning awakening) loaded on the somatic factor, whereas item 4 (sleeping too much) loaded on the motivation factor. This difference is likely attributable to variations in study populations. In older adults with major depressive disorder, hypersomnia is a core symptom and is associated with greater illness severity, suicidality, and treatment resistance [38]. In contrast, among individuals with cognitive decline, the relevance of sleeping too much (or hypersomnia) appears to depend on the stage of cognitive impairment. A longitudinal study in individuals with SCD found that total sleep time was significantly lower in the SCD group than in the non-SCD participants [39], suggesting that sleeping too much is not a typical feature in the early stages of cognitive decline. Supporting this, Zawar et al. [40] incorporated daytime sleepiness (or sleeping too much) into a composite measure of nighttime behaviors and found that the prevalence of these behaviors increased with the severity of cognitive impairment: approximately 14% in NC, 21% in MCI, and 29% in dementia. The also noted that daytime sleepiness reflects circadian disruption that develops as neurodegeneration advances. Since this study focused on individuals with SCD and MCI—stages in whom sleeping too much is uncommon—this item showed low prevalence and limited discriminatory power. Consequently, it was excluded from the factor analysis.

Item 15 (concentration/decision-making) had the second-highest symptom prevalence in this study, after middle insomnia. Despite this, it was excluded from the factor analysis because its factor loading was below 0.5. The classification of this item has varied across previous studies. Wardenaar et al. [21] included it in the mood/cognition factor. An Indonesian study [22] assigned it to the anxiety/somatic factor. Hegeman et al. [37] reported cross-loadings on both the mood and motivation factors, leading to the exclusion of item 15. Although attention deficits are a core symptom of major depressive disorder and demonstrate good sensitivity in depressed populations, this study focused on individuals with SCD and MCI. In this context, the applicability of this item may differ. We propose that attention and decision-making, as assessed by this item, reflect a subjective evaluation of executive function. In participants with SCD and MCI, this item fits neither the physiological domain of the anxiety/somatic factor nor the affective-cognitive domain of the mood/cognition factor. For instance, one study reported that among adults aged 58 to 98 years, the ability to sustain attention and control impulses increased until around 75 to 80 years [41]. This suggests that in individuals with SCD, self-reported difficulties in attention and decision-making may reflect heightened sensitivity to perceived cognitive changes rather than actual cognitive decline [42]. 

In this study, HAMD and the HAMA were used as reference standards to evaluate the criterion validity of the IDS-SR in individuals with SCD and MCI. The results showed that both the total score and subscale scores of the IDS-SR were significantly and positively correlated with HAMD and HAMA scores. These findings indicate good criterion validity in the early stages of cognitive impairment and are consistent with previous studies [43]. The correlation coefficients were highest in the cognitively normal group, followed by the SCD group, and lowest in the MCI group. This pattern may reflect the impact of cognitive function on the accuracy of self-report. As cognitive impairment worsens, deficits in insights, memory, and expression may reduce reporting accuracy. Previous studies have also indicated that cognitive impairment can weaken the validity of self-report measures in this population [44]. Notably, the correlation between the anxiety/somatic subscale of the IDS-SR and HAMA was higher in the SCD group than in the MCI group. This difference may relate to how individuals perceive cognitive decline. People with SCD tend to overestimate their deficits, whereas those with MCI may underestimate or more accurately assess their impairment [45]. Overall, the IDS-SR remains a valid tool for assessing mood symptoms in early cognitive impairment. However, its measurement precision may decline as cognitive deficits increase.

This study also examined the correlations between the IDS-SR and two cognitive measures, the ACE‑III and the MoCA‑B. The results showed that the total IDS‑SR score was negatively correlated with the ACE‑III score. This finding indicates that more severe depressive symptoms are associated with poorer cognitive performance. It is consistent with previous studies [46, 47]. One explanation is that depressive symptoms, such as psychomotor slowing and inattention, directly affect performance on cognitive tests. Tartt et al. [48] proposed that depression is associated with structural brain changes, neuroinflammation, and dysregulation of neurotransmitter systems. These changes may disrupt hippocampal neurogenesis and neuroplasticity, which can lead to hippocampal atrophy and accelerated cognitive decline. Notably, no significant correlation between IDS‑SR scores and the MoCA‑B scores was found. This result may relate to impaired emotional recognition in this population. Such impairment can reduce the ability to recognize report emotional states accurately [49]. As a result, participants may underreport or deny symptoms when completing the IDS-SR. Another explanation is that the MoCA-B may better distinguish levels of cognitive impairment than the ACE-III. Its scores may be less affected by depressive symptoms.

In this study, the Cronbach’s α for the total IDS-SR scale was 0.839, and the values for the subscales ranged from 0.670 to 0.786. These results indicate good internal consistency in individuals with SCD and MCI. These findings are comparable to those of Rush et al. [20], who reported Cronbach’s α ranging from 0.92 to 0.94. They are also consistent with the range of 0.70 to 0.93 reported by Hegeman et al. [37] for subscales in older adults with major depressive disorder. The ICC was 0.952, indicating good temporal stability in this early-stage dementia population. This result is higher than that reported in previous studies. For example, one study reassessed 24 patients after a one-week interval and reported a test-retest reliability of 0.718 [50]. More recently, the IDS-SR has been used to evaluate treatment outcomes in pharmacotherapy studies for depression, which further supports its stability [51].

Depression and anxiety often overlap in older adults [52]. ROC analysis showed that the IDS-SR has good diagnostic accuracy for identifying depression with anxiety. This advantage over single-dimensional scales—such as Patient Health Questionnaire-9 (PHQ-9)—further underscores its ability to capture the complex and overlapping symptoms. The optimal cut off score for distinguishing the depressive symptoms was 12.5 in the SCD group and 14.5 in the MCI group. The difference may be related to cognitive function status. Specifically, individuals with MCI often exhibit more severe cognitive impairment and may present with more prominent mood symptoms. Notably, previous studies have not established a unified cut-off for the IDS-SR. Rush et al. [20] proposed a cutoff of 18 for clinically significant depression in patients with major depressive disorder. Another study adopted a cutoff 14 to identify depressive symptoms in patients with prostate cancer [53]. The cutoff scores identified in this study fall between these values, suggesting that the optimal cutoff may vary with the cognitive status of the target population.

The IDS-SR has a distinct advantage in its comprehensive coverage of both depressive and anxiety symptoms. In contrast, GDS, CES-D, and PHQ-9 do not address certain symptom domains. The GDS is widely used for assessing depression in older adults. It is efficient to administer, ease to score, and has favorable psychometric properties [54]. It items mainly reflect affective symptoms (e.g., sadness, apathy, and crying) and cognitive symptoms (e.g., thoughts of hopelessness, helplessness, guilt, and worthlessness) [18]. However, it does not assess somatic concerns commonly seen in older adults (e.g., changes in energy, appetite, sleep, and sexual interest) [18]. Similarly, the CES-D does not assess the diagnostic criteria such as loss of appetite, apathy, psychomotor changes, guilt, or suicidal tendencies [18]. These symptoms are particularly important in patients whose depressive symptoms are expressed somatically. The PHQ-9 is used to assess and monitor depression severity [55]. While it exhibits strong specificity, it also lacks coverage of somatic symptoms and does not capture broader negative emotional states [56]. The IDS-SR compensates for these limitations through its item design. In this study, the most common initial 16 items of IDS-SR are listed in Table S2. Among these, somatic symptoms such as “middle insomnia”, and “aches and pains” were highly prevalent. Somatic symptoms, including medically unexplained physical symptoms, are an important issue in primary care. Many older adults report physical symptoms without identifiable pathological findings, which can reflect underlying psychiatric disorders such as anxiety or depression [57]. Compared to younger adults, older adults have a higher prevalence of somatic symptom disorders [58], partly due to higher rates of comorbidities. Cultural factors also play a role. In Chinses populations, patients with depression often focus on physical pain rather than emotional problems [59]. A sense of shame around expressing mood symptoms can lead individuals to present emotional distress as physical discomfort [60]. This tendency can lead to underdiagnosis of mood disorders and misses opportunities for appropriate treatment. Therefore, a comprehensive assessment of both somatic and psychological symptoms is essential. The IDS-SR address this need, which distinguishes it from other commonly used scales.

### Limitations

This study has several limitations. First, its cross-sectional design limits the generalizability of the findings. The data capture mood symptoms at a single time point and do not reflect changes over time. Second, although a senior psychiatrist made clinical diagnoses according to DSM IV criteria, the study did not use structured clinical interviews. Future studies incorporating structured interviews would provide a more rigorous confirmation of these results. Third, although we conducted CFA in the MCI group to validate the factor structure, we did not perform formally test measurement invariance across different subgroups. Future studies are warranted to examine multi-group measurement invariance.

## Conclusions

In summary, the IDS-SR scale demonstrates good validation and accuracy for assessing mood disorders in individuals with SCD and MCI, particularly in identifying depression with anxiety. The scale is suitable for both clinical and research applications. However, its effectiveness may be influenced by cognitive impairment. Further research is needed to validate these findings.

## Supplementary Information

Supplementary Material 1.

Supplementary Material 2.

## Data Availability

All data and study materials are available from the corresponding author, Qihao Guo, on reasonable request.

## Abbreviations

SCD: Subjective cognitive decline
MCI: Mild cognitive impairment
HRSD: Hamilton Rating Scale for Depression
HAMD: Hamilton Depression Rating Scale
HAMA: Hamilton Anxiety Scale
GDS: Geriatric Depression Scale
DSM IV: Diagnostic and Statistical Manual of Mental Disorders, Fourth Edition
CSDD: Cornell Scale for Depression in Dementia
IDS-SR: The Inventory of Depressive Symptomatology Self-Report
CES-D: Centre for Epidemiologic Studies Depression Scale
QIDS-SR: The Quick Inventory of Depressive Symptomatology Self-Report
C-PAS: Chinese Preclinical Alzheimer's Disease Study
ACE-III: Addenbrooke’s Cognitive Examination III
MoCA-B: Montreal Cognitive Assessment-Basic
ANOVA: One-way analysis of variance
EFA: Exploratory factor analysis
CFA: Confirmatory factor analysis
KMO: Kaiser-Meyer-Olkin
PCA: Principal component analysis
χ2/df: Chi-square/degree of freedom
RMSEA: Root mean square error of approximation
CFI: Comparative fit index
TLI: Tucker-Lewis index
IFI: Incremental fit index
GFI: Goodness of fit index
AGFI: Adjusted goodness-of-fit index
ROC: Receiver operating characteristic
AUC: Area under the curve
ICC: Intraclass correlation coefficient
CI: Confidence interval
NC: Normal cognition
PHQ-9: Patient Health Questionnaire-9
