# Supplementary material for: Validity of the Inventory of Depressive Symptomatology Self-Report (IDS-SR) in Older Adults With Subjective Cognitive Decline (SCD) and Mild Cognitive Impairment (MCI)
Source: BMC Psychol. 2026 May 11;14:1438. doi: 10.1186/s40359-026-04514-5 (PMC13640320; doi:10.1186/s40359-026-04514-5)
Supplement: Supplementary file 2 [file 40359_2026_4514_MOESM2_ESM.docx]

**Table S1 All IDS-SR 30 items**

**IDS-SR items**

1. Initial insomnia

2. Middle insomnia

3. Early morning awakening

4. Sleeping to much

5. Feeling sad

6. Feeling irritable

7. Feeling anxious or tense

8. Reactivity of mood

9. Diurnal variation of mood

10. Quality of mood

11. Decreased Appetite

12. Increased Appetite

13. Decreased Weight (Within the last two weeks)

14. Increased Weight (Within the last two weeks)

15. Concentration/decision-making

16. Self criticism and blame

17. Future pessimism

18. Suicidal thoughts

19. Interest in people/activities

20. Energy/fatigability

21. Pleasure or enjoyment (not sex)

22. Interest in sex

23. Psychomotor retardation

24. Psychomotor agitation

25. Aches and pains

26. Sympathetic arousal

27. Panic/phobic symptoms

28. Constipation/diarrhoea

29. Interpersonal sensitivity

30. Leaden paralysis/Physical energy

**Note:** IDS-SR: Inventory of Depressive Symptomatology Self-Report.

**Table S2 The prevalence of each of the first 16 items in IDS-SR scale (N=1670)**

| **IDS-SR items** | **N** | **%** |
| --- | --- | --- |
| IDS-SR 2 | 1125 | 67.37 |
| IDS-SR 15 | 823 | 49.28 |
| IDS-SR 25 | 798 | 47.78 |
| IDS-SR 22 | 792 | 47.43 |
| IDS-SR 23 | 755 | 45.21 |
| IDS-SR 1 | 747 | 44.73 |
| IDS-SR 6 | 698 | 41.80 |
| IDS-SR 20 | 674 | 40.36 |
| IDS-SR 26 | 671 | 40.18 |
| IDS-SR 7 | 661 | 39.58 |
| IDS-SR 3 | 633 | 37.90 |
| IDS-SR 30 | 558 | 33.41 |
| IDS-SR 5 | 470 | 28.14 |
| IDS-SR 28 | 450 | 26.95 |
| IDS-SR 17 | 384 | 22.99 |
| IDS-SR 27 | 317 | 18.98 |

**Abbreviations:** IDS-SR: Inventory of Depressive Symptomatology Self-Report.
